# Postoperative cerebral hemorrhage death in a patient with secondary hyperparathyroidism: a report of one case and literature review

**DOI:** 10.3389/fnins.2023.1153453

**Published:** 2023-05-11

**Authors:** Peng Zhou, Jing Xu, Dayong Zhuang, Xiaolei Li, Tao Yue, Huaiqiang Hu, Qingqing He

**Affiliations:** ^1^Department of Thyroid and Breast Surgery, The 960th Hospital of People's Liberation Army, Jinan, China; ^2^Department of Neurology, The 960th Hospital of People's Liberation Army, Jinan, China

**Keywords:** secondary hyperparathyroidism, end-stage renal disease, hemorrhagic stroke, parathyroid surgery, perioperative management

## Abstract

Secondary Hyperparathyroidism (SHPT) is a common complication of end-stage renal disease (ESRD), and parathyroid surgery (PTX) is an effective way to treat patients with severe SHPT. ESRD has multiple associations with cerebrovascular diseases. For example, the incidence of stroke in patients with ESRD is 10 times higher than that in the general population, the risk of death after acute stroke is three times higher, and the risk of hemorrhagic stroke is significantly higher. High/low serum calcium, high PTH, low serum sodium, high white blood cell count, previous occurrences of cerebrovascular events, polycystic kidney disease (as a primary disease), and the use of anticoagulants are independent risk factors for hemorrhagic stroke in hemodialysis patients with uremia. The risk of stroke in patients who undergo PTX decreases significantly in the second year of follow-up and persist thereafter. However, studies on the risk of perioperative stroke in SHPT patients are limited. After undergoing PTX, the PTH levels in SHPT patients drop suddenly, they undergo physiological changes, bone mineralization increases, and calcium in the blood gets redistributed, often accompanied by severe hypocalcemia. Serum calcium might influence the occurrence and development of hemorrhagic stroke at various stages. To prevent bleeding from the operated area, the use of anticoagulants after surgery is reduced in some cases, which often decreases the frequency of dialysis and increases the quantity of fluid in the body. An increase in the variation in blood pressure, instability of cerebral perfusion, and extensive intracranial calcification during dialysis promote hemorrhagic stroke, but these clinical problems have not received enough attention. In this study, we reported the death of an SHPT patient who suffered a perioperative intracerebral hemorrhage. Based on this case, we discussed the high-risk factors for perioperative hemorrhagic stroke in patients who undergo PTX. Our findings might help in the identification and early prevention of the risk of profuse bleeding in patients and provide reference for the safe performance of such operations.

## 1. Introduction

Stroke is the main cause of disability and death in hemodialysis (HD) patients. The chances of stroke are several times higher in HD patients than that in the general population, especially because cerebral hemorrhage has a huge impact on patient outcomes (Kelly et al., 2021). Secondary hyperparathyroidism (SHPT) is a common and serious complication in patients with chronic renal failure, and parathyroidectomy is the main way of treating SHPT patients who are refractory to medical treatment (Lau et al., 2018; Choi et al., 2021). Various dialysis methods, comorbidities, and drugs can affect platelet aggregation and/or coagulation cascade and, thus, affect the homeostasis of bleeding and hemostasis. The level of serum calcium, high PTH, low serum sodium, high leukocyte count, and a history of cerebrovascular events are important factors that influence hemorrhagic stroke in patients with hemodialysis. Several clinical studies have investigated the pathogenesis of CKD-MBD stroke, along with its prevention and treatment (Xiao et al., 2017; Yang et al., 2022). After undergoing PTX due to PTH, SHPT patients experience many physiological changes. The perioperative usually strictly control fluid intake because the changes in calcium homeostasis, dialysis, and the effect of multiple factors, such as antihypertensive drugs, blood pressure fluctuations, vascular tone, blood-brain barrier changes, and the risk of hemorrhagic stroke, might increase (Wakasugi et al., 2015). Few studies have investigated the prevention and treatment of perioperative stroke in patients after PTX. In this study, we reported the death of a patient due to cerebral hemorrhage after she underwent PTX. We analyzed the cause to provide a reference for increasing the safety of such surgeries. We present the following case following the CARE reporting checklist.

## 2. Case description

**Figure 1:**
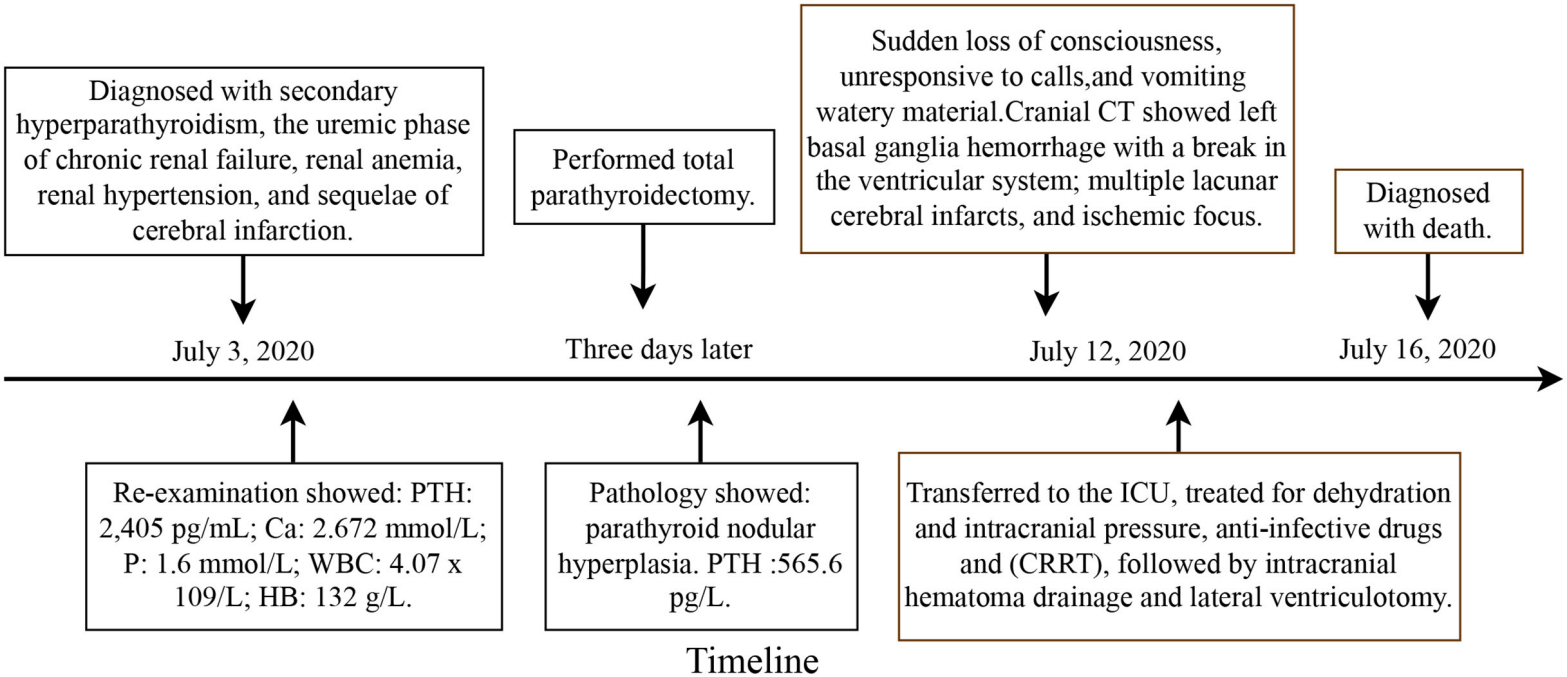
The timeline of case details.

In 2020, a patient with SHPT expired due to cerebral hemorrhage after parathyroid surgery in our unit. The details regarding this case are as follows and Figure 1 is showcasing a timeline with relevant data from the episode of care. The patient was a 51-year-old female who was admitted to the hospital with uremic dialysis for 9 years and elevated parathyroid hormone for more than 5 years. She was suffering from hypertension for more than 10 years, epilepsy for more than 9 years, and cerebral infarction for more than 2 years. She was taking various oral medications. On admission, the patient suffered from bone pain in extremities with limited mobility and pruritus all over the skin, and no other uncomfortable physical symptoms. During admission, she was diagnosed with secondary hyperparathyroidism, the uremic phase of chronic renal failure, renal anemia, renal hypertension, and sequelae of cerebral infarction. After admission, a thyroid ultrasound examination showed posterior nodules in both lobes of the thyroid gland, which were considered to be of parathyroid origin. Re-examination showed the following: PTH: 2,405 pg/mL; Ca: 2.672 mmol/L; P: 1.6 mmol/L; WBC: 4.07 x 10^9^/L; HB: 132 g/L. Total parathyroidectomy was performed on July 7, 2020, and postoperative pathology showed (upper right, lower right, upper left, and lower left) parathyroid nodular hyperplasia. PTH decreased to 565.6 pg/L on intraoperative examination, and blood calcium and phosphorus decreased to the normal range on the first day after surgery. The clinical symptoms of the patients, such as bone pain with limited mobility and skin pruritus, improved significantly after surgery. On the morning of July 12, 2020, the patient suddenly became unconscious (deep coma with a GCS score of 3). She was unresponsive to calls and vomited a large quantity of watery material. Her pupillary reflex to light was poor, and emergency cranial CT showed left basal ganglia hemorrhage with a break in the ventricular system; multiple lacunar cerebral infarcts, and ischemic focus. The neurosurgeon evaluated the intracranial hemorrhage by CT imaging at about 75 ml. The patient was transferred to the intensive care unit, where she was treated for dehydration and intracranial pressure and was administered anti-infectives and continuous renal replacement therapy (CRRT), followed by intracranial hematoma drainage and lateral ventriculotomy (hematoma drainage 50 ml). Cranial CT reexamination showed the area of high density in basal ganglia was reduced. Re-examination showed the following: WBC: 13.99 x 10^9^/L; PLT: 124 x 10^9^/L; D-dimer: 1.00 mg/L; Ca: 1.87 mmol/L; P: 0.60 mmol/L. The patient was resuscitated twice during hospitalization (continuous infusion of norepinephrine and vasopressin by intravenous pump) and was in a deep coma, mechanically ventilated (respirator: SIMV mode, VT 480 ml, PEEP 4 cmH2O), and without spontaneous respiration. The patient's family refused to continue using the ventilator and stopped further treatment.

## 3. Discussion

Secondary hyperparathyroidism is a common complication of end-stage renal disease (ESRD). Such patients mostly have multiple comorbidities. The overall mortality rate of patients with renal diseases, such as uremia, is 15–20% per year. Cerebral hemorrhage is ~3.8 times more common in ESRD patients than in the general population (Thome et al., 2019). According to the US Renal Data System, 3.1% of dialysis patients died of a stroke in 2011 (Collins et al., 2012). With the advancements in hemodialysis technology, the survival time of uremic patients has increased substantially. However, the incidence of SHPT is increasing, and more patients are opting for parathyroidectomy. According to the latest edition of the AAES guidelines (2022), PTX can reduce cardiovascular and all-cause mortality in patients with SHPT. The benefits of surgery significantly exceed the drawbacks in patients who do not respond to medication and have significant symptoms (Dream et al., 2022). To prevent perioperative cerebral hemorrhage or reduce its severity, a comprehensive understanding of the risk factors for cerebral hemorrhage in HD patients with modifiable factors is necessary.

### 3.1. Risk factors for concurrent cerebral hemorrhage

The risk of perioperative cerebral hemorrhage increases due to the application of anticoagulants such as heparin during dialysis, making this treatment method life-threatening. Hyperlipidemia, hypertension, diabetes mellitus, age on dialysis, and mean arterial pressure are high-risk factors for the development of complications of cerebral hemorrhage (Lau et al., 2018). Patients with a history of combined hyperlipidemia, hypertension, and diabetes mellitus may have atherosclerosis of intracranial vessels, which in turn might increase the fragility of intracranial vessel walls. Patients with advanced uremia may have increased blood volume and extracellular fluid and increased renin-angiotensin activity, resulting in water and sodium retention. This can cause an increase in mean arterial pressure, progressive aggravation of atherosclerosis, and an increase in vascular fragility. Sharp blood pressure fluctuations might cause dysregulation of the autonomic nervous system, which can lead to arterial and microaneurysm rupture and affect prognosis (Xiao et al., 2017; Yang et al., 2022). Several studies have investigated blood pressure control after CH; however, data on blood pressure before CH are limited, particularly on the increase in blood pressure before CH. Age on dialysis is strongly associated with the development of cerebral hemorrhage; the longer the age on dialysis, the higher the frequency of dialysis, which in turn gradually decreases the functions of the patient's organs (Kitamura et al., 2020). Administering exogenous anticoagulants causes fibrinogen to bind to platelets in the body, which can stimulate the production of platelet antibodies, induce thrombocytopenia, and cause bleeding. The greater the cerebral hemorrhage, the worse the prognosis. Brain tissue edema is more pronounced, and intracranial pressure is higher in patients on maintenance dialysis for uremia, and the prognosis is extremely poor if the volume of cerebral hemorrhage is >30 mL (Kitamura et al., 2019). Additionally, a decrease in hemoglobin and albumin levels increases hemorrhage and affects prognosis. The changes in hemoglobin levels after cerebral hemorrhage might be related to the higher incidence of hematoma enlargement in the early stages of a cerebral hemorrhage. Conversely, hemoglobin levels might reflect the severity and prognosis of a cerebral hemorrhage. A decrease in albumin levels can lower the resistance of the body, increase the risk of infection, and thus, aggravate the disease. Hypoproteinemia can decrease the plasma colloid osmotic pressure, promote the retention of a large quantity of fluid in the interstitial space, decrease the effective circulating blood volume, and decrease the perfusion of vital tissues and organs surrounding the site of intracranial hemorrhage, leading to a poor prognosis (Xiao et al., 2017; Kitamura et al., 2019; Yang et al., 2022). Additionally, high serum calcium, low serum sodium, high leukocyte count, a history of cerebrovascular diseases, primary polycystic kidney disease, and daily application of warfarin are independent risk factors for cerebral hemorrhage in uremic patients on regular hemodialysis.

Serum calcium can play multiple roles in each stage of the occurrence and progression of cerebral hemorrhage, as high blood calcium levels in patients with SHPT cause severe calcification of the vascular wall and trigger cerebral hemorrhage through several mechanisms (Xiao et al., 2017). Hypocalcemia is also associated with cerebral hemorrhage. Kitamura et al. showed that asymptomatic blood pressure increased in HD patients before cerebral hemorrhage; this increase in blood pressure was associated with frequent occurrences of lower serum calcium levels, but the exact mechanism of this phenomenon is unknown (Dandapat et al., 2019; Kitamura et al., 2020). In the perioperative period of parathyroidectomy (PTX), the patient is in a state of “calcium starvation” due to a sudden drop in PTH and an increase in skeletal mineralization (Liu et al., 2020), which leads to fluctuations in blood pressure due to sudden changes in multiple factors in the internal environment; these changes might promote cerebral hemorrhage. In patients with hemorrhagic stroke, moderate-to-severe CKD is associated with a 2.3-fold increase in the hematoma volume and a poor prognosis (Molshatzki et al., 2011; Nayak-Rao and Shenoy, 2017). Postoperative hypocalcemia should be aggressively treated by administering calcium supplements *via* oral and intravenous modalities.

### 3.2. Prevention and management of a cerebral hemorrhage in the perioperative period of parathyroid surgery

The hypercoagulable state of the blood in patients with SHPT increases the risk of hemorrhage. Before patients undergo surgery, cerebral hemorrhage and bleeding at the operative site should be prevented to reduce the risk of perioperative bleeding. Due to the multifactorial nature of cerebral hemorrhage, prevention and treatment options might include one or a combination of the following: dialysis, erythropoietin, cold precipitation, anticoagulation, antihypertensive drugs, and postsurgical correction of hypocalcemia (Hedges et al., 2007).

Patients should be examined as comprehensively as possible after admission. Besides the examinations related to the surgical area, examination of the cardiac, cerebral, and pulmonary conditions should be conducted to exclude contraindications to surgery. Preoperative precise localization is needed to decrease the operating time and the discomfort to the patient. Those receiving low-molecular heparin can be switched to heparin-free dialysis within 24 h before the operation. In recent years, the administration of sodium citrate as an anticoagulant in hemodialysis for patients with uremic cerebral hemorrhage has shown significant advantages. Through extracorporeal local anticoagulation without aggravating bleeding, it can ensure adequate hemodialysis, replenish blood sodium, reduce cerebral edema, promote hemorrhage absorption, and also reduce blood calcium due to the physical and chemical properties of sodium citrate. This can further reduce blood pressure and reduce inflammation and oxidative stress (Xun et al., 2021). The treatment can also be switched to anticoagulant dialysis with sodium citrate within 24 h before and after the operation. Hypertension in HD patients can prevent the risk of death, but the optimal predialysis blood pressure range remains undetermined. The risk of a new onset of cerebrovascular and arteriovenous fistula infarction increases with a decrease in blood pressure during the perioperative period. Additionally, the risk of cerebral hemorrhage decreases with better perioperative blood pressure control to reduce blood pressure fluctuations. According to the 2022 edition of the Expert Consensus, perioperative blood pressure should be regulated at a level of <180/100 mmHg (Dream et al., 2022). Since PTX was introduced in our hospital in 2010, only this patient died due to a postoperative cerebral hemorrhage. Although the risk of a cerebral hemorrhage is high, surgery can largely relieve clinical symptoms and improve the quality of life of the patients. Most patients benefit from surgery, and the incidences of patients with SHPT opting for surgery have increased.

### 3.3. Treatment and prognosis of a concurrent cerebral hemorrhage

If a cerebral hemorrhage occurs, treatment options and prognosis depend on several factors. For example, the treatment of patients with cerebral hemorrhage in uremia is divided into conservative medical treatment and surgical treatment. Surgery is the primary method for treating cerebral hemorrhage and is also the best option for patients on hemodialysis. However, the choice of surgical treatment and the type of procedure used remains undetermined. In most studies, mortality and disability rates after surgical treatment of cerebral hemorrhage were similar to those of patients treated non-surgically. Kim et al. (2013) reported three deaths in six surgically treated patients, and the other three were severely disabled. Therefore, patients with the uremic syndrome should be informed of the risks and adverse effects of surgery by neurosurgeons. (1) Surgery might be considered for patients with hemorrhage >35 mL in the basal ganglia or 50–60 mL in the cerebral lobes, but only for those who are stable and have no significant increase in hematoma on cranial CT reexamination; (2) surgery is generally not considered for patients with excessive hemorrhage (e.g., >100 mL) or patients >70 years; (3) preoperative and postoperative treatment should include active dialysis and preoperative or intraoperative transfusion of plasma, cold precipitation, or platelets to improve coagulation. Mannitol is a volumetric dehydrating agent that increases blood volume and relies on renal excretion. It can easily cross the fragile blood-brain barrier into the brain parenchyma and aggravate brain edema. To reduce intracranial pressure, glycerol fructose or albumin can be used instead of mannitol. In our study, the patient was a middle-aged female who had a combination of hypertension, cerebral infarction, and various underlying diseases. This led to a poor prognosis and increased the risk of death after the onset of a cerebral hemorrhage.

The reason for this is that ESRD is closely related to the occurrence of a cerebral hemorrhage in the perioperative period in patients with SHPT. Before patients with SHPT undergo surgery, surgeons should perform preoperative preparations and risk assessment predictions. The patients and their families should be informed of their condition and the risks and complications of surgery. In case of complications of a cerebral hemorrhage, timely diagnosis of the patients and early selection of appropriate treatment modalities might decrease their mortality and disability rate.

## Data availability statement

The raw data supporting the conclusions of this article will be made available by the authors, without undue reservation.

## Ethics statement

The studies involving human participants were reviewed and approved by the Ethics Committee of the 960th Hospital of People's Liberation Army. The patients/participants provided their written informed consent to participate in this study. Written informed consent was obtained from the participant/patient(s) for the publication of this case report.

## Author contributions

QH and PZ: contributed to the design and conception of this report. PZ and JX: data curation and writing-original draft preparation. QH, DZ, and XL: supervision. QH and TY: writing-reviewing and editing. All authors read and approved the final manuscript.
