# Supplementary material for: Postoperative cerebral hemorrhage death in a patient with secondary hyperparathyroidism: a report of one case and literature review
Source: Front Neurosci. 2023 May 11;17:1153453. doi: 10.3389/fnins.2023.1153453 (PMC10213765; doi:10.3389/fnins.2023.1153453)
Supplement: Supplementary file 1 [file Table_1.DOCX]

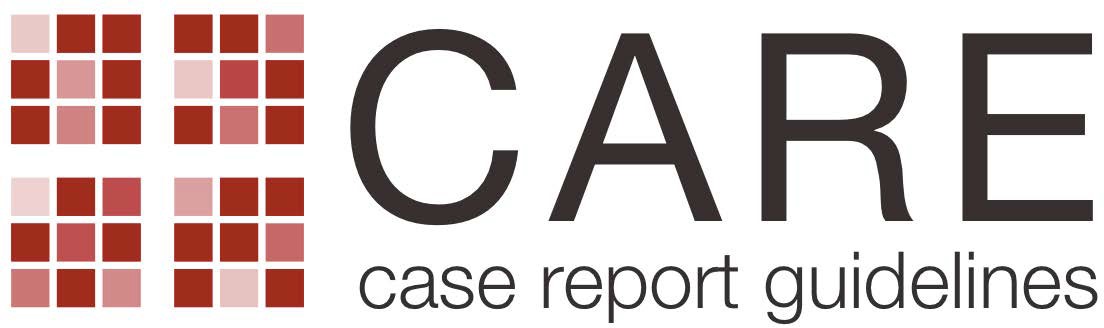
CARE Checklist of information to include when writing a case report
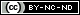


**Topic Item Checklist item description Reported on Line**

**Title 1** The diagnosis or intervention of primary focus followed by the words “case report” 1

**Key Words 2** 2 to 5 key words that identify diagnoses or interventions in this case report, including "case report" 17

**Abstract**

**(no references)**

**3a** Introduction: What is unique about this case and what does it add to the scientific literature? 22

**3b** Main symptoms and/or important clinical findings 33

**3c** The main diagnoses, therapeutic interventions, and outcomes 38

**3d** Conclusion—What is the main “take-away” lesson(s) from this case? 39

**Introduction 4** One or two paragraphs summarizing why this case is unique (**may include references**) 43

**Patient Information 5a** De-identified patient specific information 67

**5b** Primary concerns and symptoms of the patient 78,80

**5c** Medical, family, and psycho-social history including relevant genetic information 68

**5d** Relevant past interventions with outcomes 75,83

**Clinical Findings**

**Timeline**

**Diagnostic Assessment**

**Therapeutic Intervention**

**Follow-up and Outcomes**

1. Describe significant physical examination (PE) and important clinical findings 74,82
2. Historical and current information from this episode of care organized as a timeline 66

**8a** Diagnostic testing (such as PE, laboratory testing, imaging, surveys). 76,82

**8b** Diagnostic challenges (such as access to testing, financial, or cultural) 80

**8c** Diagnosis (including other diagnoses considered) 82

**8d** Prognosis (such as staging in oncology) where applicable

**9a** Types of therapeutic intervention (such as pharmacologic, surgical, preventive, self-care) 83

**9b** Administration of therapeutic intervention (such as dosage, strength, duration) 84

**9c** Changes in therapeutic intervention (with rationale) 82

**10a** Clinician and patient-assessed outcomes (if available)

**10b** Important follow-up diagnostic and other test results 86

**10c** Intervention adherence and tolerability (How was this assessed?) 88

**10d** Adverse and unanticipated events 86

**Discussion 11a** A scientific discussion of the strengths AND limitations associated with this case report 89

**11b** Discussion of the relevant medical literature **with references** 89

**11c** The scientific rationale for any conclusions (including assessment of possible causes) 103,152,183

**11d** The primary “take-away” lessons of this case report (without references) in a one paragraph conclusion 178,191,204

**Patient Perspective 12** The patient should share their perspective in one to two paragraphs on the treatment(s) they received

**Informed Consent 13** Did the patient give informed consent? Please provide if requested . . . . . . . . . . . . . . . . . . . . . . . . . . . . . . . . . . . . . . **Yes ☑ No**
